# Perioperative rational thromboprophylaxis strategy contributes to reduce adverse clinical effects of delayed total hip arthroplasty

**DOI:** 10.3389/fmed.2025.1602605

**Published:** 2025-07-04

**Authors:** Fangshi Xu, Zongyu Li, Hao Guan, Jiancang Ma

**Affiliations:** Department of Vascular Surgery, The Second Affiliated Hospital of Xi'an Jiaotong University, Xi'an, Shaanxi, China

**Keywords:** femoral neck fracture, total hip arthroplasty, deep vein thrombosis, venous thromboembolism, anticoagulation

The recent research entitled “Trends and benefits of early hip arthroplasty for femoral neck fracture in China: a national cohort study” demonstrates the great clinical benefit of early total hip arthroplasty (THA) in elderly patients with femoral neck fractures (FNFs) (1). In particular, early THA gains distinctly competitive advantages over delayed surgery in the field of the prevention of pulmonary embolism (PE) and deep vein thrombosis (DVT). Unfortunately, according to the data from Chinese national electronic inpatient database, <20% of patients received early THA therapy. Nonetheless, delayed THA does not mean the catastrophic outcomes. Based on the perspective of vascular surgery, we argue that the preoperatively accurate assessment of venous thromboembolism (VTE) risk and reasonable strategy of DVT prevention will greatly reduce the risk of PE and DVT in patients receiving delayed THA, thereby mitigating the adverse effects brought by delayed surgery (Figure 1).

**Figure 1:**
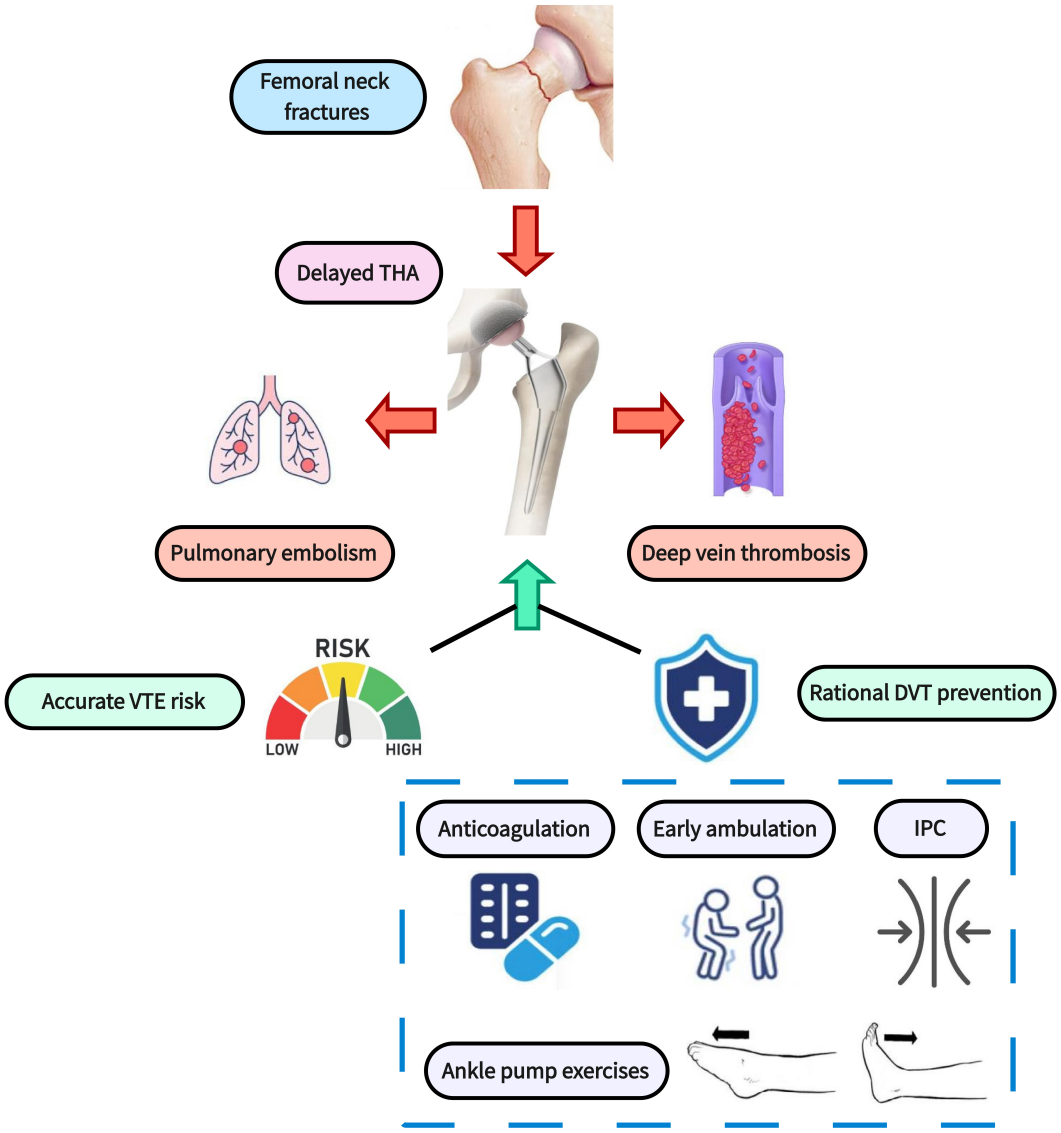
Perioperative rational thromboprophylaxis strategy for patients receiving delayed THA. THA, total hip arthroplasty; VTE, venous thromboembolism; DVT, deep vein thrombosis; IPC, intermittent pneumatic compression.

## Emphasize preoperative VTE risk assessment

PE and DVT are crucial factors that affect the prognosis of FNFs patients. According to statistics, over 9.4% of inpatients are accompanied by DVT and thus face the threat of PE. Therefore, it is important to conduct timely and precise assessments of VTE risk and use it as the foundation for developing a reasonable prevention program. There are two critical decision points in this procedure. First, Caprini score should be performed on all FNFs patients prior to surgery. Currently, this grading scale is the most commonly used system for assessing thrombotic risk in surgical inpatients, with higher scores denoting greater DVT risks (2). Luksameearunothai et al. (3) demonstrated that FNFs patients with a Caprini score higher than 12 had a 7.56 times greater risk of DVT than patients with a score lower than 12. For elderly FNFs patients, they are commonly concomitant with advanced age (2 points), prolonged immobilization (2 points), long operation time (2 points), and broken hip or pelvis (5 points), totaling 11 points. Therefore, patients require extra concern when they simultaneously present with other common risk factors, such as obesity, chronic obstructive pulmonary disease, active cancer, atrial fibrillation, and varicose veins in the lower extremities, as these cases would have the scores >12. These patients should be defined as high-risk for VTE and appropriate thrombus prophylaxis needs to be taken into account.

Secondly, greater focus should be placed on the dynamic assessment and re-scoring of patients with delayed THA. The alterations of D dimer, leg circumference, and respiratory status of FNFs patients, especially for those receiving delayed surgery, deserve extra attention. For instance, if a patient manifests with a high or persistently elevated D dimer level during waiting times to surgery, prompt anticoagulation is generally beneficial. Recently, Li et al. (4) developed a model based on D-dimer levels and platelet distribution width for predicting the incidence of DVT in patients with femoral neck. In this predictive model, the odd ratio (OR) of high D-dimer levels (Age^*^0.02 mg/L) was as high as 2.718, highlighting the potential significance of early anticoagulant interventions.

## Preoperative reasonable DVT prophylaxis

Anticoagulation is fundamental and critical to DVT prevention. Nevertheless, balancing the benefits of anticoagulation and the risk of bleeding in clinical practice remains a challenging endeavor. As for patients with high risk for VTE, preoperative anticoagulation is commonly safe and essential. For instance, the 2024 edition of the guideline for perioperative cardiovascular management for non-cardiac surgery based on American Heart Association pinpoints that patients with high risk for thrombotic complications should be treated with preoperative bridging of anticoagulant therapy (5). Moreover, a study from Mayo Clinic revealed that only 0.3% of surgical patients who received preoperative anticoagulation suffered from fatal bleeding events (6). Of course, the risk of bleeding associated with anticoagulation is an issue that cannot be ignored. Although controversial, a study indicated that perioperative antithrombotic drugs had a greater than two-fold increased likelihood of developing a significant postoperative bleed (7). Therefore, the development of an anticoagulation regimen for patients receiving delayed THA should be based on an accurate VTE risk assessment, and multidisciplinary collaboration from vascular surgery is particularly important.

Notably, except for anticoagulant therapy, early ambulation, ankle pump exercises, and intermittent pneumatic compression (IPC) are also essential parts for DVT prevention. For instance, a meta-analysis confirmed that although IPC combined with pharmacologic prophylaxis was not more preferable in DVT prevention than single pharmacologic prophylaxis, was superior in bleeding prevention (OR = 0.17). These non-anticoagulant approaches are especially important for patients with a high risk of bleeding (8).

## Conclusions

Due to insufficient healthcare resources and the limitations of medical technology, most elderly patients with FNFs in China are unable to receive THA intervention at the early stage, and delayed treatment exposes these patients to the threat of DVT and PE, which greatly and adversely affects the prognosis of these elderly trauma patients. Faced with this grim situation, timely and accurate VTE risk assessment and a rational anticoagulation prophylaxis regimen based on it will hopefully alleviate the thrombotic risk associated with delayed THA.
